# Gentamicin, genetic variation and deafness in preterm children

**DOI:** 10.1186/1471-2431-14-66

**Published:** 2014-03-05

**Authors:** Maria Bitner-Glindzicz, Shamima Rahman, Kathy Chant, Neil Marlow

**Affiliations:** 1 Genetics and Genomic Medicine, University College London Institute of Child Health and Great Ormond Street Hospital for Children, 30 Guilford Street, London WC1N 1EH, UK; 2 Neonatology Department, University College London Institute for Women’s Health, Medical School Building, 74 Huntley Street, London WC1E 6 AU, UK

**Keywords:** Gentamicin, Aminoglycosides, Deafness, Hearing loss, Mitochondrial, m.1555A > G, Preterm, Prematurity

## Abstract

**Background:**

Hearing loss in children born before 32 weeks of gestation is more prevalent than in full term infants. Aminoglycoside antibiotics are routinely used to treat bacterial infections in babies on neonatal intensive care units. However, this type of medication can have harmful effects on the auditory system. In order to avoid this blood levels should be maintained in the therapeutic range. However in individuals with a mitochondrial genetic variant (m.1555A > G), permanent hearing loss can occur even when drug levels are within normal limits. The aim of the study is to investigate the burden that the m.1555A > G mutation represents to deafness in very preterm infants.

**Method:**

This is a case control study of children born at less than 32 completed weeks of gestation with confirmed hearing loss. Children in the control group will be matched for sex, gestational age and neonatal intensive care unit on which they were treated, and will have normal hearing. Saliva samples will be taken from children in both groups; DNA will be extracted and tested for the mutation. Retrospective pharmacological data and clinical history will be abstracted from the medical notes. Risk associated with gentamicin, m.1555A > G and other co-morbid risk factors will be evaluated using conditional logistic regression.

**Discussion:**

If there is an increased burden of hearing loss with m.1555A > G and aminoglycoside use, consideration will be given to genetic testing during pregnancy, postnatal testing prior to drug administration, or the use of an alternative first line antibiotic. Detailed perinatal data collection will also allow greater definition of the causal pathway of acquired hearing loss in very preterm children.

## Background

Advances in neonatal intensive care have resulted in substantial improvements in outcome, in particular the survival of very preterm infants [1]. However, among the surviving population there is a high level of disability [2,3]. The prevalence of deafness is around 1-2% in preterm or low birth weight babies, and is often co-morbid with other disabilities such as cerebral palsy, and intellectual and visual impairment [4]. Hearing loss in this group of infants is up to ten times more prevalent than in term babies [4]. Deafness has long term implications for communication skills, educational achievement, and overall quality of life, even in mild cases of unilateral and bilateral hearing loss [5]. The cause of acquired deafness in this group is likely to be multifactorial although several high risk factors have been suggested. These include very low birth weight, severe jaundice [6], hypoxia, medication [7] and infection [8].

Infection is a common occurrence during the neonatal period due to the underdeveloped immune system of the preterm infant, and can rapidly become life threatening. Aminoglycoside antibiotics are frequently used as a first line of treatment for suspected or proven bacterial sepsis. High efficacy, low levels of resistance and low cost make this group of medications an effective and recommended option [9]. Aminoglycoside exposure is known to have nephrotoxic and ototoxic side effects, hence regular monitoring of blood concentrations. However reassurance has been taken from the repeated observations that aminoglycoside toxicity does not appear to be a frequent occurrence in this population.

A genetic predisposition renders some individuals more susceptible to rapidly progressive, permanent hearing loss even when aminoglycoside levels are maintained within the therapeutic range. The most common predisposing mutation is the mitochondrial m.1555A > G variant, a maternally inherited genetic trait. Although the exact process of aminoglycoside induced deafness is unknown it is thought that the m.1555A > G mutation enables aminoglycosides to bind to the mitochondrial ribosomes more readily than the normal genetic sequence. This is thought to result in abnormal translation of mitochondrial proteins necessary for energy production. Hair cells in the inner ear are metabolically highly active and consequently have large numbers of mitochondria. In addition, aminoglycosides easily enter the hair cells but take a long time to be cleared [10].

In susceptible individuals, a single dose of aminoglycoside may cause hearing loss. Early indications were that penetrance of deafness following aminoglycoside exposure was close to 100% in the presence of m.1555A > G. For example, in a study of European families with a history of maternally transmitted deafness and presence of the m.1555A > G mutation, all individuals who received aminoglycosides became deaf [11]. Furthermore, there was a lower mean age of onset of deafness in the group exposed to these antibiotics implicating aminoglycosides as a catalyst for rapidly progressive hearing loss. However, it is possible that such research is biased. Individuals that are given aminoglycosides with no ill effect are unlikely to be genetically screened for the mutation. More recently in identical twins born at 24 weeks gestation both with m.1555A > G and treated with aminoglycosides, only one of the twins has hearing loss. The deaf twin received multiple courses of aminoglycosides whilst the twin with normal hearing received a single course (unpublished data). The hearing twin showed no clinical indication for genetic screening, but was tested with the other twin. It is possible that single courses may be tolerated in some cases.

The Avon Longitudinal Study of Parents and Children cohort (ALSPAC) showed the prevalence of the mutation to be 1 in 520 children [12]. Audiological screening was normal at the age of 9 years, there were no histories of admissions to neonatal units and exposure to aminoglycosides was thought to be unlikely. Thus, there were no clinically distinguishing features between those with the mutation and those without, and therefore no reason under normal circumstances for genetic testing. Nevertheless, it is also possible that there had been exposure to ototoxic medication but hearing had been preserved, which would indicate that the penetrance of aminoglycoside-mediated ototoxicity in m.1555A > G is less than previous research has suggested. These findings demonstrate the need for further research in this domain.

The primary aim of this study is to evaluate the contribution of the m.1555A > G mutation to acquired deafness in babies born at a gestational age of less than 32 weeks, who are likely to have received multiple doses of aminoglycoside antibiotics. Further to this, the study aims to calculate the relative odds of carrying the genetic variant and being in the deaf group versus the hearing group. Risk associated with gentamicin, m.1555A > G and other co-morbid risk factors will be evaluated using conditional logistic regression to determine potential gene-drug interactions in the context of other ototoxic risks.

## Method/Design

### Study design

Case control study, with 5 controls for each index case.

### Definition of index cases

Any child born at 31 weeks and 6 days gestation or less with confirmed hearing loss (>40dBHL). Children with clear genetic syndromic causes for hearing loss will be excluded.

### Index population

This will include all children with hearing loss who were born at less than 31 weeks and 6 days gestation between January 2009 and December 2013. All children will have been treated in a Neonatal Intensive Care Unit (NICU) within the Greater London region. Participants will be identified through electronic databases, hearing assessment centres, neonatal follow up services and advertisements promoting the study.

### Control population

Each child recruited with hearing loss will be matched with five children with normal hearing. Control children will be identified through the Standardised Electronic Neonatal Database (SEND) which contains demographic and clinical information about infants admitted to neonatal units across London. Matching will be based upon gestational age (same number of completed weeks of gestation), sex (male/female) and neonatal unit on which they received their neonatal intensive care over the first two weeks after birth. Control children with partially complete or missing pharmacological records will be excluded on the premise that other matched control children will be identifiable with more comprehensive records.

### Procedure

Index children will be invited to participate in the study via a letter sent by the audiological paediatrician responsible for their care. This will enclose information about the study and parents who require further information or would like to participate will be asked to return a short form to indicate their willingness to speak to the research team. The research nurse will telephone the parent(s), explain the study further and arrange a convenient time to meet parents if they wish to participate. Parents who identify themselves to the research team via newsletters or advertisements will be spoken to directly by the research team. Written informed consent will be obtained for genetic testing of a saliva sample, and to extract data from neonatal and audiological clinical notes. During a subsequent home visit saliva samples will be taken if testing for m.1555A > G has not been undertaken as part of clinical care. Saliva samples will be collected using an Oragene collection kit and transported to the laboratory for testing. DNA is extracted using standard procedures and analysed by polymerase chain reaction and restriction enzyme digestion, followed by sequence confirmation by bi-directional Sanger sequencing in mutation-positive cases.

Matched controls will be identified though the national neonatal research dataset. Parents of control children identified will be invited to participate by letter sent by the clinician responsible for their neonatal care. Those wishing to participate in the study will contact the research team as above.

Children identified to have the mutation will be recalled and the presence of m.1555A > G reconfirmed by analysis of DNA extracted from a second sample. Families confirmed to have the mutation will be offered genetic counselling. Since mitochondrial DNA is maternally inherited, there are additional implications for extended maternal family members, who will also be offered genetic counselling.

Clinical data will be extracted from the neonatal and audiological medical notes for all children using an agreed proforma to record details of demographics, clinical events and therapeutic history, with serum levels of drugs as available. Data will be collected by day after birth to identify coincidence of risk factors. Pharmacy records will be examined to confirm aminoglycoside exposure, including dose, duration, number of courses and blood level concentrations. Audiological records will be reviewed to define the severity and pattern of hearing loss.

Data will be encoded for data analysis using double entry into an automated error detection system in SPSS (IBM Inc) in its latest version. Subsequently data will be checked for outliers, prior to analysis using Stata (Version 13).

### Sample size

The annual birth-rate in London is approximately 120 000, (600 000 in 5 years). It is estimated that 1% of these babies have a birth weight of less than 1501 g [13]. This gives a likely population of babies at less than 32 weeks gestational age of 3 000–6 000 over 5 years. The prevalence of hearing loss within this group is 1-2% [4], predicting 30–120 children with hearing loss over the 5 year study period. From a cohort of 3 000–6 000 children, we anticipate that 6–12 children will have the m.1555A > G mutation, given a mutation frequency of 1 in 526 (95% CI 1 in 357 to 1 in 770) [14]. For each index child, we will recruit up to 5 matched controls.

Thus the total study population should include between 180–720 children, 30–120 index and 150–600 controls.

### Statistical analysis

The burden of disease will be calculated as the proportion (and 95% CI) of children with hearing loss that have the mutation, using the method of Wilson. The odds ratio of children with the m.1555A > G mutation having hearing loss will be calculated using Fisher’s exact test. The analysis for the burden of disease and the relative risk of deafness for those with the mutation will initially include all cases. This will be repeated excluding children who received less than one dose of aminoglycoside medication, and repeated again excluding those who received fewer than two doses.

The antecedents and associates of hearing loss will be determined using conditional logistic regression to account for matching. Factors will be evaluated individually and as combinations occurring simultaneously to account for additive risk.

### Ethical issues

The study has been approved by the National Research Ethics Service (NRES Committee London – Central, ref: 12/LO/0005) and is registered under the National Institute for Health Research (NIHR) Portfolio scheme. The study is funded by a financial grant from Action on Hearing Loss; the funding source has had no input into the design or execution of the research study.

## Discussion

We aim to investigate the contribution of m.1555A > G to deafness in preterm infants where aminoglycosides are likely to have been used and to evaluate this role within the occurrence of multiple risk factors for hearing loss identified during neonatal intensive care. We hypothesise that the mutation will make a significant contribution to deafness even when aminoglycoside levels are maintained within the therapeutic range. From our clinical experience, we suspect that in some cases, single courses of aminoglycosides may be tolerated but multiple courses may not be, possibly due to the accumulation of the medication in the hair cells of the inner ear, which can take several months to clear. Multiple courses of aminoglycosides during the neonatal period would thus inhibit the clearance process.

Alongside such a potential interaction, to date multiple studies have failed to provide consistent evidence for specific risk factors. Intriguingly one small study has reported that it is the coincidence of risk factors that may determine the development of hearing loss [4]. A range of factors, such as other ototoxic drugs, hyperbilirubinaemia, acidosis and sepsis, will be evaluated as part of this study and these risks assessed for co-occurrence. The conditional case control analysis will maximise the potential sensitivity of this large study to determine the presence of such associations.

Should m.1555A > G be implicated in the aetiology of neonatal hearing loss in very preterm babies, then alternative strategies may be necessary to manage preterm neonatal sepsis in affected babies.

Alternative first line antibiotics could be used instead of aminoglycosides. Using more broad spectrum agents runs the risk of increasing patterns of bacterial resistance in the relatively closed microbiological setting of neonatal intensive care. Further, owing to low cost and the synergistic effects of aminoglycosides, their use is preferred by many services for babies at risk of life threatening infection, considering that the risk of aminoglycoside associated hearing loss is as yet unsubstantiated in newborn babies.

A further alternative would be universal genetic screening (of pregnant women) or focussed genetic screening of babies prior to treatment, which is at present impracticable [15]. Due to the nature of illness experienced by many babies being treated on the neonatal intensive care unit, aminoglycoside administration may be required before the results of genetic screening are available. Screening women during pregnancy would overcome this since the mutation is maternally inherited but this is expensive and would require clear evidence that this interaction is a key step in the causal pathway. This would, however, enable early recognition of the potential for hearing impairment and lead to the use of an alternative medication for these infants.

*Mitogent* is designed to provide critical answers concerning the aetiology of acquired hearing impairment in very preterm babies and specifically to identify the role of aminoglycoside toxicity in individuals with m.1555A > G.

## Abbreviations

ALSPAC: Avon longitudinal study of parents and children cohort; dBHL: Decibels hearing level; NICU: Neonatal intensive care unit; SEND: Standardised electronic neonatal database; CI: Confidence interval; NIHR: National institute for health research.

## Competing interests

The authors declare that they have no competing interests.

## Authors’ contributions

KC wrote the first draft and produced the final version. NM, SR and MB-G conceived the idea and obtained funding. Each has reviewed this paper and approved the final version.

## Authors’ information

KC is the research fellow conducting the study; NM is a professor of neonatal medicine and has a major interest in perinatal influences on long term outcomes; SR is a Reader and Consultant in metabolic and mitochondrial medicine; MB-G is a professor of clinical and molecular genetics specialising in genetic hearing loss.

## Pre-publication history

The pre-publication history for this paper can be accessed here:

http://www.biomedcentral.com/1471-2431/14/66/prepub
